# Understanding lifetimes and failure modes of defective washing machines and dishwashers

**DOI:** 10.1016/j.jclepro.2019.01.044

**Published:** 2019-04-01

**Authors:** Paolo Tecchio, Fulvio Ardente, Fabrice Mathieux

**Affiliations:** European Commission, Joint Research Centre (JRC), Ispra, Italy

**Keywords:** Lifetime, Durability, Repair, Circular economy, Failure mode

## Abstract

The available literature on average lifetimes and failure modes of household appliances is mainly based on results of surveys conducted among end-users, but very little precise information can be found on specific failure modes and repair rates.

The main objective of this study is to provide quantitative data about frequent failures and average service lifetimes of two household appliances, through the analysis of repair services performed by professional repair operators.

We based our analysis on available datasets provided by a representative independent repair centre based in Europe, and we focused on the failures most frequently occurring and the potential repair or discard of the appliances. A database of about 11,000 diagnoses on defective washing machines and dishwashers was analysed, and frequent failure modes and repair rates were identified. The analysis was supported by a tailored visualisation of results.

Concerning washing machines, recurring failures diagnosed by the repair operator regarded the electronics, shock absorbers and bearings, doors, carbon brushes and pumps. While the highest repair rates (repaired devices over total diagnosed devices with a specific failure mode) were observed for doors, carbon brushes and removal of foreign objects, the lowest rates were observed for bearings, drums and tubs, circulation pumps and electronics. Regarding dishwashers, recurring failures involved pumps, electronics, aquastop and valves, foreign objects and doors. The lowest repair rates, however, were again observed for circulation pumps and electronics. We also observed that the average service lifetime of an appliance not repaired by repair centre operators is 12.6 years for washing machines and 12 years for dishwashers.

This work brings important knowledge on lifetimes and failure modes of defective washing machines and dishwashers, concerning in particular weak and critical components, but also age of appliances to be repaired. Based on the exercise on the two appliances, we discuss a possible classification scheme for repair services of household appliances, including both information retrieved by professional repair operators and information retrieved through interviews with end-users.

## Highlights

• Repair is a recommended practice for circular economy but specific knowledge on product groups lacks.
• More than 11,000 diagnoses on defective washing machines and dishwashers were analysed.
• Failure mode categories were specified and correlated to various parameters.
• A tailored visualisation of data provides insights about the service lifetime of the two appliances.
• A classification of repair services of household appliances was formulated.

## 1 Introduction

Expected lifetime of products is key information to estimate the stock of materials in products and potential end-of-life (EoL) flows. In the raw material area, product durability is recognised to have the potential to play a key role in enhancing resource efficiency, as recalled by the European Raw Materials Initiative in its third pillar (European Commission, 2008). Indeed, durable products allow an efficient use of resources over time, reducing the consumption of raw materials and contributing to waste minimization (Bocken et al., 2016). Also the Strategic Implementation Plan of the European Innovation Partnership on raw materials highlighted the need for work on product life extension strategies (European Commission, 2013). Thus, product durability is a main product design strategy to address material efficiency (Allwood and Cullen, 2012; Ghisellini et al., 2015) and several studies confirmed that environmental benefits can be gained by extending the lifetime of products, such as: washing machines (Ardente and Mathieux, 2014), dishwashers (Tecchio et al., 2016), vacuum cleaners (Bobba et al., 2016), personal computers (Bakker et al., 2014b; Prakash et al., 2016b, 2012; Tecchio et al., 2018), refrigerators (Bakker et al., 2014b) and other small electric and electronic equipment (Bovea et al., 2017).

However, the actual lifetime of products, such as mobile phones, televisions, washing machines, coffee machines and dishwashers, is often perceived as short by end-users and not always matching their expectations (Hennies and Stamminger, 2016; Wieser and Tröger, 2016). Furthermore, according to a 2014 Eurobarometer survey, 77% of the European consumers would rather repair their goods than buy new ones, but ultimately have to replace or discard them because they are discouraged by the cost of repairs and the level of service provided (European Commission, 2014).

In spite of this, extending lifetime of products is currently a high priority for industries and policy makers, as proved by the European Circular Economy action plan that promises to “emphasise on circular economy aspects in future product requirements under the Ecodesign directive” (European Commission, 2015a). The absence of adequate metrics and standards has been a key barrier to the inclusion of resource efficiency requirements, such as durability, in Ecodesign measures (Tecchio et al., 2017a). However, European standardisation bodies are currently working to develop standards on how assessing product durability, reparability and reusability, thanks to the mandate M/543 issued by European Commission in the context of the Ecodesign Directive (CEN-CENELEC, 2018; European Commission, 2015b).

To support these activities with solid and scientific evidences, we analysed different references providing estimations of the durability and average lifetimes of household appliances, whose figures originated from surveys among end-users. However, we identified a lack of precise information on frequent failure modes, their causes and the potential aptitude of users to (or not to) repair or discard an appliance. Extensive analyses of repair activities outside the warranty period time are also lacking. Nonetheless, investigating the product lifetimes based on questionnaire surveys that rely on respondents’ memory would not provide precise results (Oguchi and Daigo, 2017). Instead, repair records stored by professional repair operators represent a huge source of information concerning these aspects. Thus, our strategy is to use information from repair operators to better understand lifetimes of products, including the reasons to discard a product.

The main objective of this study is to provide quantitative data about frequent failures and to test whether the analysis of professional repair services of a representative European repair operator can bring valuable knowledge on product lifetime of these two product groups. The database of repair services consists of a total of about 11,000 datasets on defective washing machines and dishwashers. The analysis of the database led us to describe the most recurring failure modes (including information about the type of failure mode, repair actions, replacement of components, reasons not to repair), and the correlation with service lifetimes. Finally, we draw recommendations on how to classify repair services of household appliances and how to improve future work.

## 2 Scientific background

The average lifetime of household appliances, such as washing machines and dishwashers, is dependent upon many factors: stress, abrasion, maintenance, technological change, fashion, shift in values and other external environmental influences (Prakash et al., 2016a). Considering the overall resource efficiency of an energy related product, the appliance should be durable enough to achieve the service lifetime expected by end-users. Nonetheless, the service lifetime should also be appropriate to technological progress and energy performance of newer products, as most of the significant environmental impacts depend on the energy consumption during the use phase (Bundgaard et al., 2017).

Several references for average washing machine and dishwasher lifetimes can be found in the literature. Concerning washing machines, Prakash et al. (2016a) reported 11.6 years as the average first useful service-life of washing machines in Germany. Hennies and Stamminger (2016) analysed the responses of another survey among German users and observed that washing machines are discarded when their age reaches 12 years old, on average, but variations are largely influenced by the purchase price and usage frequency. Bakker et al. (2014a, 2014b), based on another survey among Dutch users, also observed that the median lifespans of washing machines has reduced over time, from 12.1 years in 2000 to 11.7 years in 2005. In regards to dishwashers, similar figures can be observed. Considering the Swedish market, Johansson and Luttropp (2009) reported a life expectancy ranging from 10 to 15 years. Prakash et al. (2016a) reported 12.4 years as the average first useful service-life of dishwashers in Germany. As such, the average lifetime considered for these two appliances in the preparatory studies on Ecodesign requirements was set to 12.5 years, in both cases (Boyano Larriba et al., 2017a, 2017b).

According to Hennies and Stamminger (2016), who analysed a total of 1,075 interviews obtained via an internet-based questionnaire of end-users, the rich amount of data about washing machines designates the appliance as representative of white goods. According to the respondents, washing machines are discarded: 1) if they are defective, 2) if the machine is no longer liked, 3) if the machine is not satisfying because of its features, 4) if the device is not resource-efficient enough, 5) if the appliance is replaced by a present or 6) for other reasons. In 69% of cases the reason for the disposal of washing machines was a defect (Hennies and Stamminger, 2016). Also, 90% of washing machine sales are primarily due to replacements after product failures (VHK, 2014). As a consequence, many household products are simply treated as waste rather than being repaired and/or reused, resulting in a negative consequence for the environment (McCollough, 2009).

From the analysed literature it was observed that end-users generally expect these products to last longer. Other products, such as mobile phones or televisions are replaced relatively rapidly, as end-users may decide to upgrade and replace an item before the end of its working life. This is not the case with washing machines (Ricardo-AEA, 2014a, 2014b), and white goods in general. Two possible strategies to extend the lifetime of washing machines and dishwashers consist of the reduction of failure frequency and the increased reparability. The definition of priority parts, such components, assemblies, or any other hardware or software constituents which are important for the repair/upgrade of the product, is also a key element (Cordella et al., 2018). However, there is no simple and straightforward solution to this issue. The authors of the present article already worked on approaches to contribute to the goal of ‘extending lifetime’ of products, in previous work. Tecchio et al. (2017a) proposed a framework to boost sustainable engineering and resource use by systematically identifying standardisation needs and features. The framework was tested on a case study on washing machine durability, identifying specific standardisation needs, adequate metrics for performance measurements, reliable and repeatable tests, and calculation procedures. Specific guidelines on how to develop (possible) standardised durability tests were then proposed by Stamminger et al. (2018), who developed and tested an endurance procedure for the whole product, under conditions of stress. The goal of the endurance procedure was to ensure minimum durability requirements and to reduce the failure frequency of the whole device. However, further research is needed to define the criteria and the conditions for a repeatable, relatively fast and relevant endurance tests applicable to the whole product group.

Another approach to extend the lifetime of products is to increase their reparability in case of defect. While obsolescence is not only an issue that should be addressed to the manufacturer, but also as a responsibility to the consumer (Hennies and Stamminger, 2016), product durability can be facilitated by the extended availability of spare parts for replacement in the event of repair (ONR, 192102, 2014). A detailed list of technical barriers to repair for the two product groups was already published by Deloitte (2016): 1) technical barriers (functional obsolescence, software updates and short innovation cycles); 2) economic barriers (uncertainties regarding the guarantee of the repair service; small price differences between the repair and the purchase of a new product may make repair and reuse unattractive) and 3) legal barriers (manufacturers and retailers are not always obliged to provide consumers or the repair market with technical instructions, the expected technical lifetime of the product or the availability of spare parts). In particular, repair operators are identifying the lack of instructions and technical information availability as the key obstacle to the repair of fridges, dishwashers and washing machines (RREUSE, 2013). Nonetheless, the ease of disassembly of products varies depending on the initial design of the product and on how different product parts can be further separated into single components (Peeters et al., 2018; Vanegas et al., 2018).

To the knowledge of the authors, very little precise information can be found on frequent failure modes and repair rates of household appliances. Prakash et al. (2016a) reported the results of lifetime studies conducted by Stiftung Warentest on washing machines over timeline 2000–2014. Tests involved a total of 600 appliances and 196 different models, of which 41 of them encountered problems during the test for a 10-year usage. RREUSE (2013) reported a series of washing machine failures due to low quality of materials and components. Low quality shock absorbers and ball bearings can fail if exposed to high spin speeds and thus affect the service lifetime of washing machines (the replacement of ball bearings often required the purchase of a complete washing unit, whose cost is comparable to a new product). Other reported problems related to quality of materials and reason of early failures concern the rubber of sealants and the membrane of pressure switches (Tecchio et al., 2017b). Finally, consumer organizations analysed survey results (at the European level) and indicated doors, drain pumps and spinning function as the most occurring failures for washing machines (Altroconsumo, 2015; OCU, 2015).

## 3 Method

While previous work on failure modes and average lifetimes of products were mainly based on surveys among end-users (Bakker et al., 2014b; Cox et al., 2013; Gnanapragasam et al., 2017; Hennies and Stamminger, 2016), we based our analysis on available datasets from a professional repair operator. The independent repair centre was the Austrian Reparatur-und Service-Zentrum (RUSZ), which is based in Vienna and active on the creation of networks of repair operators. RUSZ deals with thousands of appliances every year, and supported this analysis by providing a database of repair services.

Our research approach consisted of two main steps. As a first step, RUSZ was asked to provide a database of repair services performed on washing machines and dishwashers over the years 2009–2015. RUSZ provided a database of more than 11,000 datasets, in which each dataset reports a diagnosis on a defective washing machine or dishwasher. Thanks to its procedure for product diagnosis, RUSZ could provide detail on each repair service, such as the type of appliance, the detected failure mode(s), the repair activities (e.g. including when an appliance required the replacement of one or more parts, use of new or second-hand spare parts, etc.) or reasons not to repair the defective appliance. Thus, we were able to conduct an analysis of the main information that can be extracted from such a database, namely: 1) temporal distribution of device diagnosed; 2) classification of devices diagnosed with a single failure mode versus devices diagnosed with multiple failure modes (i.e. more than one failure observed on the same appliance); 3) classification of recurrent failure modes (see section 4.1), detail on repaired and unrepaired devices; 4) main reasons not to repair a device (see section 4.2); 5) focus on principal failure modes and/or most-repaired components.

As a second step, we narrowed the analysis to a subset of repair services occurring in the first quarter of 2016. This part of the database was also populated with more detailed information collected to better classify appliances at the moment of failure. We specifically asked RUSZ to interview end-users to retrieve information on the defective appliance. Specifically, end-users were asked to detail the age of the device at the moment of the repair service, the average use rate by the user (i.e. washing cycles/week), and the number of previous repair services (if any). The 2016 database is made up of a total of 428 repair services on washing machines and 262 on dishwashers. In 396 cases (255 washing machines and 141 dishwashers) the customer was able to answer the three questions mentioned above.

The second goal of this further analysis was also to identify possible correlation between recurring failure modes and age of products at the moment of the diagnosis, but also with usage frequency and previous repair services performed on the same appliance. For this aim, we developed tailored visualisation of results for this specific subset of data.

## 4 Results

### 4.1 Failure mode classifications for washing machines and dishwashers

One of the first analyses performed on the database was to classify the different failure modes reported by RUSZ, in order to handle a limited number of classifiers. Each dataset, indeed, informs on which specific failure mode(s) or defective component(s) were diagnosed. The failure mode classification aimed to identify and group frequent failure modes, and was discussed and validated by RUSZ. The different categories are listed in Table 1.

**Table 1:** Failure mode classifications for washing machines and dishwashers. Detail of the categories used to group frequent failure modes for the two product groups.

Washing machines - failure mode categories	Dishwashers - failure mode categories
**Electronics**: includes control electronics, engine electronics, inverter electronics, relays, programs selectors or control panels, line filters, displays. **Shock absorbers and bearings**: includes shock absorbers, bearings, ball bearings. **Doors**: includes door handles, hinges, locks and seals. **Carbon brushes**. **Pumps**: includes circulation pumps and drain pumps. **Foreign objects**: includes foreign objects detected. **Drain system**: includes drain hose, outlet hoses, drain systems, inlet hoses. **Inlet valves**: includes mechanical or electronic aquastop and other inlet valves. **Switches**: includes float switches, micro switches, on-off switches, keypad. **Engine**: includes engine, engine condenser and tachogenerator. **Pump filters**. **Drive belt**: includes drive belt/pulley. **Heater and thermostats**. **Drum and tub**. **Pressure control**: includes pressure chamber, pressure control, air hoses. **Detergent system**: includes detergent drawer and hose. **Cables and plugs**: includes cables, wiring and plugs. **Other**: includes other unusual failure modes.	**Electronics**: includes control electronics, relays, sensors, program selectors, control panels, displays. **Doors**: includes door brakes, handles, hinges, locks and seals. **Pumps**: includes circulation pumps and drain pumps. **Foreign objects**: includes foreign objects detected in pumps (drain pumps mainly) and drain systems. **Drain system**: includes drain hose, outlet hoses, drain systems, inlet hoses. **Inlet valves**: includes mechanical or electronic aquastop and other inlet valves, water distributor. **Switches**: includes float switches, micro switches, on-off switches, keypad. **Engine**: includes engine, engine condenser. **Heater and thermostats**. **Pressure control**: includes pressure chamber and pressure control. **Detergent system**: includes water tank, salt container and detergent dispenser. **Spray arm**: includes: spray arms and spray arm feed pipes. **Cables and plugs**: includes cables, wiring and plugs. **Other**: includes other unusual failure modes, involving baskets, bearings, filters, program failures, tub leaky, ventilators, wheels, etc.

### 4.2 Reasons not to repair washing machines and dishwashers

Another initial analysis on the database was to classify the different reasons not to repair a defective washing machine or dishwasher. As a result, we observed three main reasons, namely: 1) “Consumer choice”, when the overall cost of spare parts and labour is regarded as too high by consumers; 2) “Technically infeasible”, when the technical barriers (such as the lack of spare parts or the ineffective design for disassembly) hamper repair; and 3) “Non-viable”, when although technically feasible the repair was judged non-viable (e.g. for functional reasons, or because the appliance was likely to fail again) and the technicians advised the consumers to discard the appliance.

We remark that customers might have had specific reasons to discard a defective appliance, however this information was not captured by technicians during repair services. It is also noted that one of the activities of the repair operator is to extract and store certain components from discarded appliances, to be used as spare parts during repair services. Replacing defective components with “second-hand” spare parts gave the possibility to lower the total repair cost. However, as this aspect was not systematically registered during the repair services, we do not provide statistics concerning new or used spare parts used for repair services.

### 4.3 Analysis of the 2009–2015 database

The database provided by RUSZ reported a total of 11,144 initial diagnoses on defective washing machines and dishwashers registered across 2009–2015. The diagnoses were performed by technicians on washing machines and dishwashers claimed to be malfunctioning. During the diagnoses, it happened that technicians did not find any failure or simply had to reset the machines from error codes; thus, these datasets (871 appliances) were classified as “no failure found” and removed from the analysis. One or more failure modes were instead detected in 6,672 washing machines and 3,469 dishwashers. In the remaining 132 cases, technicians could not detect the failure mode and did not proceed further with the repair service. Overall, 5,106 malfunctioning washing machines were successfully repaired, while 1,550 cases were not repaired by technicians due to economic or technical barriers. Regarding dishwashers, 2,503 cases were successfully completed with a repair action, while 961 appliances were again not repaired due to economic or technical barriers. For the remaining 21 appliances, only partial repairs were performed. As the reasons to perform only a partial repair was not detailed, these datasets were removed from the analysis.

**Fig. 1:**
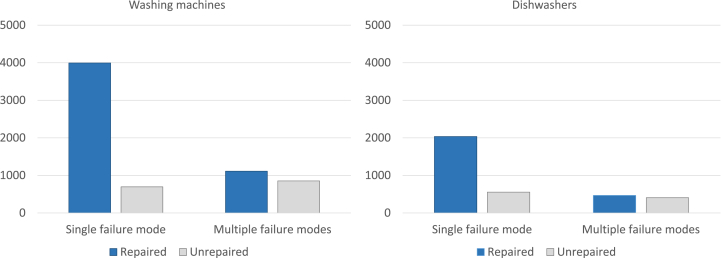
Number of repaired and unrepaired appliances, divided into *Single* and *Multiple failure modes*, *Dishwashers* and *Washing machines*.

Fig. 1 represents the breakdown between devices diagnosed with single failure mode or multiple failure modes (i.e. when more than one of the failure mode categories of Table 1 occur). It results that the latter situation is certainly more difficult to handle, depending on the type of failure mode and on the type of repair (economically feasible or infeasible; technically possible or impossible). Specifically, for the repair services involving multiple failure modes it was not always possible to identify if a failure mode triggered the other(s), nor whether there was a clear relationship between different failure modes on the same appliance.

Multiple failure modes occurred in almost 30% of diagnosed washing machines and in about 25% of diagnosed dishwashers. Appliances with multiple failure modes were not repaired in 43% of washing machine cases, and in 46% of the dishwasher cases.

**Fig. 2:**
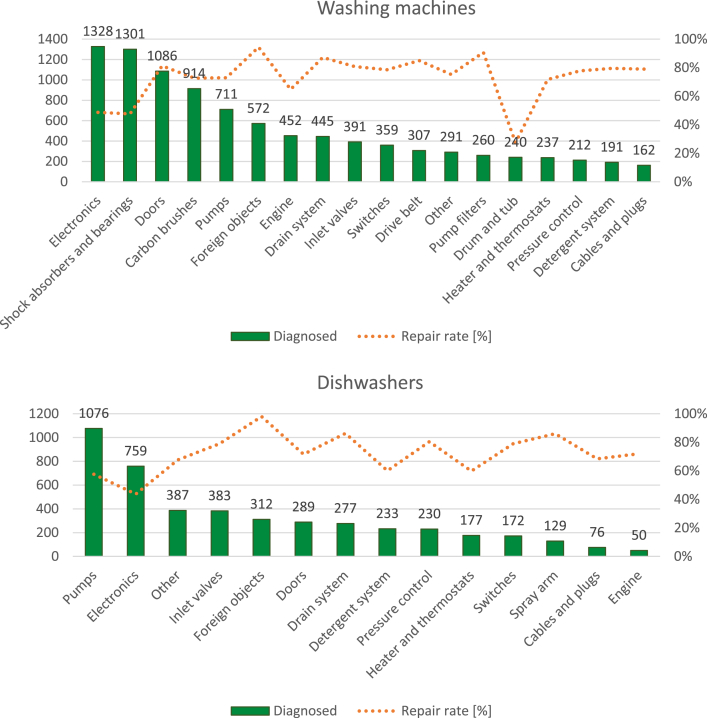
Repair rate and total number of diagnosed washing machines and dishwashers, divided by failure mode categories.

By combining single and multiple failure modes, a total of 9,492 specific failure modes were observed for washing machines, and 4,561 for dishwashers (Fig. 2). The most recurrent failure modes identified for the two appliances were related to defective components (electronics, shock absorbers and bearings, doors, carbon brushes and pumps for washing machines; pumps, electronics, inlet valves, foreign objects and doors for dishwashers). The lowest repair rates (number of repaired appliances over the total number of diagnosed appliances with a specific failure mode) for washing machines were observed when the defective component was the drum and tub (about 27% of cases), the electronics (about 49% of cases) and shock absorbers and bearings (about 47% of cases). Concerning dishwashers, the lowest repair rates were observed for pumps (about 58% of the cases) and electronics (about 44% of the cases).

**Fig. 3:**
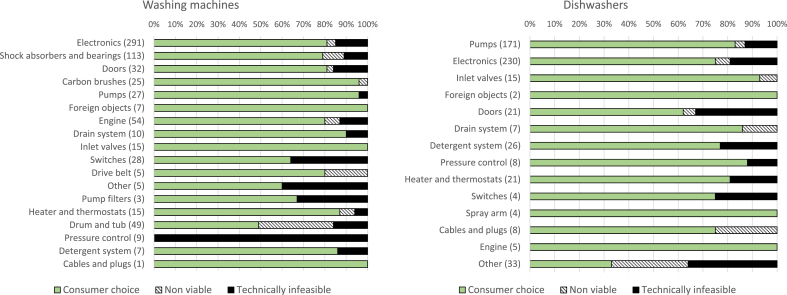
Reasons not to repair washing machines and dishwashers, divided by failure mode (values in parenthesis refer to the number of appliances considered). Values in percentage.

Narrowing the analysis to datasets with single failure modes allows to understand what were the main reasons not to repair an appliance diagnosed with a specific failure mode (Fig. 3).

By the analysis of the three main reasons not to repair a defective appliance we discovered that about 78% of unrepaired washing machines and 76% of unrepaired dishwashers were ascribable to the “Consumer choice” category. About 15% of unrepaired washing machines and 17.5% of unrepaired dishwashers were not repaired because classified as “technically infeasible”. Finally, about 7% of unrepaired washing machines and 6.5% of unrepaired dishwashers were classified as “non-viable”.

Certain failures, occurring in particular components, were more challenging to repair. Concerning dishwashers, breaks in circulation pumps were repaired in only 46% of cases, mainly due to the cost of spare parts. Especially newer circulation pumps incorporate electronic parts such as printed circuit boards and control electronics, to be able to perform the various washing programs. This results in higher costs and more challenging repairs, as it may be that the replacement of the electronic board also requires the replacement of the whole circulation pump. On the other hand, defective drain pumps were repaired in more than 72% of cases. Another low repair rate can be observed when failure occurs in electronics. Breaks in electronics were repaired only in 51% of cases. The main reason not to repair was again the overall cost, but a considerable number of technically infeasible repairs were registered for control electronics (about 19.3% of cases). The same trend can be observed for washing machines. Breaks in electronics were repaired in only 50% of cases, and the main reason not to repair was again cost, with a significant percentage of impossible repairs registered for control electronics in particular (about 10%). In this case, the accessibility of spare parts, software access and updates is key.

For washing machines, breaks in shock absorbers and bearings were repaired only in about 48% of repair services (when considering bearings only, this percentage decreases to 24%), due to the overall cost of repair and due to poor design for disassembly applied to the tub bearings. Very low rates of repaired washing machines were also observed when the defective components were the drum and tub (27%).

### 4.4 Analysis of the 2016 database

The database provided by RUSZ in 2016 (available as Supplementary material) reported a total of 690 repair services, 428 on washing machines and 262 on dishwashers. In 396 cases (255 washing machines and 141 dishwashers) the customer was able to provide additional information concerning the age of the device, the average frequency of use and the previous repair services performed on the appliance.

**Fig. 4:**
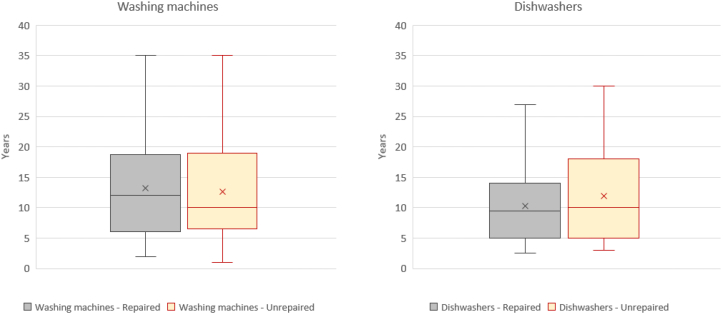
Box plots representing the distribution of the average age of the appliances at the moment of the initial diagnosis (first quartile, median, third quartile and whiskers. X represents the mean value). Distributions are divided into repaired and unrepaired dishwashers, and repaired and unrepaired washing machines.

The first analysis done on this database concerned the information on the age of the appliance at the moment of the initial diagnosis (Fig. 4).

The average age of an appliance successfully repaired by repair centre operators is 13.2 years for washing machines and 10.3 years for dishwashers (arithmetic mean values). On the other hand, the average service life of an appliance not repaired by repair centre operators is 12.6 years for washing machines and 12 years for dishwashers. Only in the case of dishwashers, it is possible to observe that an older appliance had also less probability to be repaired.

**Fig. 5:**
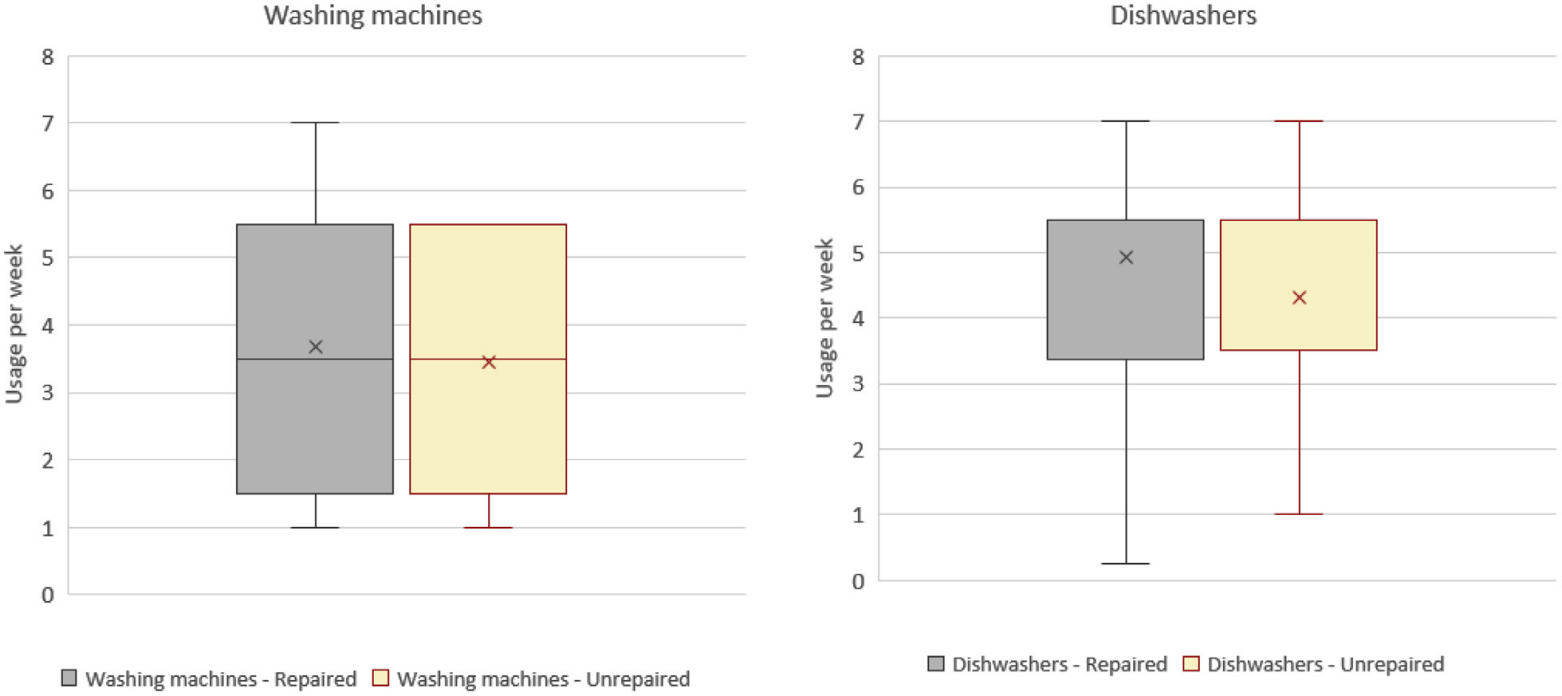
Box plots representing the distribution of the average use of the appliances (number of cycles per week) at the moment of the initial diagnosis (first quartile, median, third quartile and whiskers. X represents the mean value). Plots are divided into repaired and unrepaired dishwashers, and repaired and unrepaired washing machines.

**Fig. 6:**
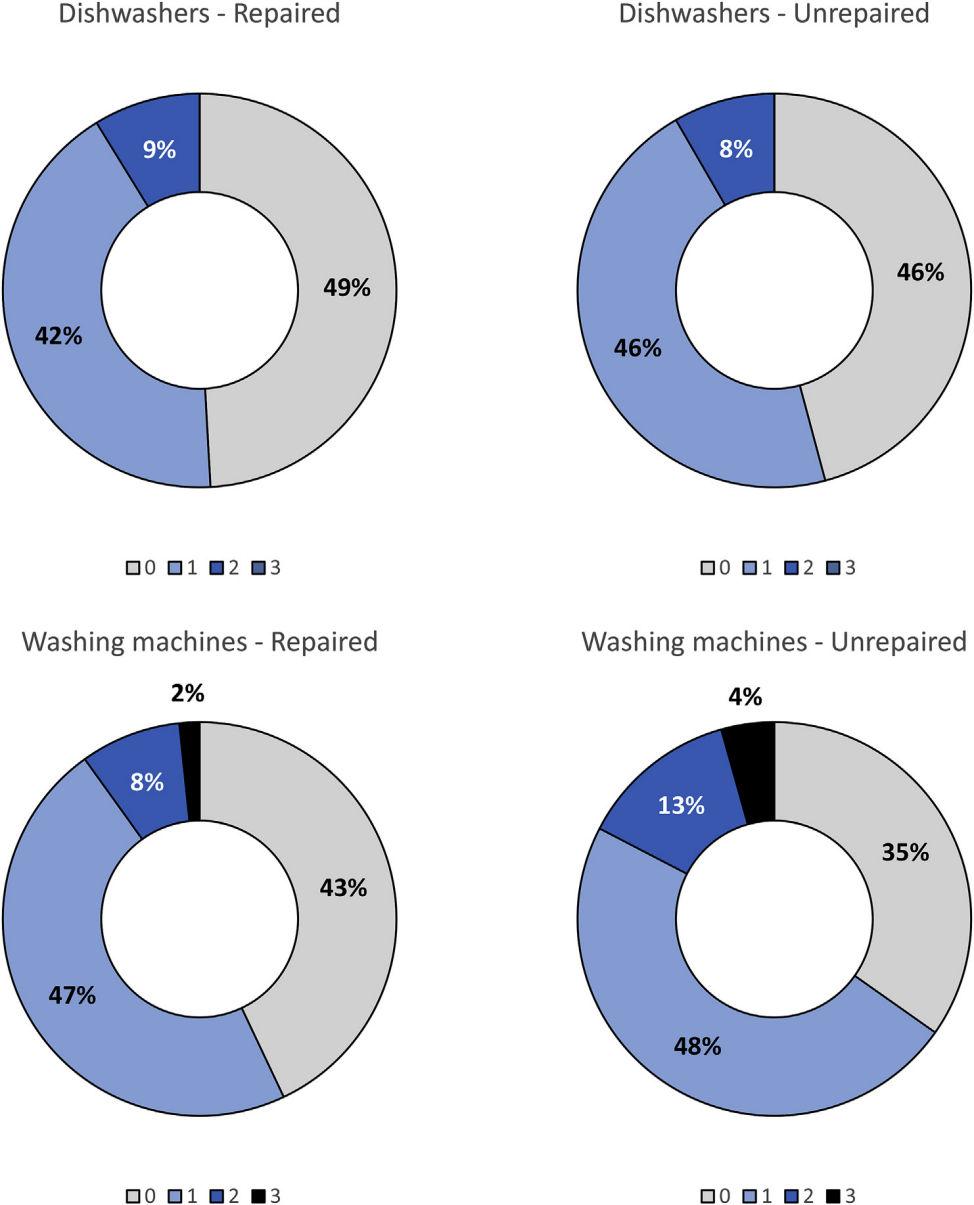
Pie charts representing the number of previous repairs (0–3) performed on the appliance at the moment of the initial diagnosis. Plots are divided into repaired and unrepaired dishwashers, and repaired and unrepaired washing machines.

We then performed a second analysis to understand if and how the other two parameters (average use of the device in washing cycles per week (Fig. 5), and number of previous repairs performed on the appliance (Fig. 6)) influence the average service life of appliances, even though it was not possible to identify clear trends. Fig. 5 presents the distributions of the average use frequency (number of cycles per week) at the moment of the initial diagnosis. On average, washing machines are used 3 to 4 times per week, while dishwashers 4 to 5 times per week. It is possible to observe that the probability of failure is not dependent on the intensity of use, for the analysed sample.

Regarding previous repairs (Fig. 6), 173 washing machines and 65 dishwashers were reported to have had already undergone some repair services before the diagnosis. Also in this case there are no clear trends or correlations between previous repairs and probability of failure, although we can confirm that the older gets the washing machine in the database, the higher is the probability that it had already undergone more than one repair.

The number of observations including new parameters monitored in 2016 was quite limited compared to the 2009–2015 database, and this may affect the distribution of results represented in Figs. 4 and 5. In detail, we found no evident correlations between the repair rates and the age of the device, the average use and number of previous repairs.

Thus, we analysed the database focusing again on the identified failure mode categories. Table 2 represents the average age of the (repaired or unrepaired) appliances, classified by failure mode. The same table also specifies the number of cases in which clients were able to provide information about the age of the appliance. The analysis was narrowed to observe the failure modes where the information about the age of the appliance was provided in at least 10 cases.

**Table 2:** Average age of the device undergoing repair services, divided by type of appliance, failure mode category and successful or unsuccessful repair. The number of diagnosed appliances by failure mode category is also specified.

Appliance	Washing machines			Dishwashers		
	Average age (years)		Diagnosed appliances^a^	Average age (years)		Diagnosed appliances^a^
Repair	Repaired	Unrepaired	*Total*	Repaired	Unrepaired	*Total*
Inlet valves	17.7	18.9	*17*	17.4	15.3	*7*
Shock absorbers and bearings	16.2	11.8	*49*	–	–	*-*
Cables and plugs	–	8.0	*1*	2.5	–	*1*
Carbon brushes	12.6	13.7	*35*	–	–	*-*
Pumps	9.5	14.2	*24*	9.0	15.0	*20*
Electronics	12.7	10.2	*19*	9.3	14.1	*22*
Door	18.7	13.5	*41*	15.0	12.8	*9*
Detergent system	23.1	12.0	*9*	11.0	17.3	*4*
Switch	5.0	13.7	*4*	–	15.0	*2*
Foreign objects	8.6	–	*25*	10.9	–	*20*
Engine	12.3	13.0	*18*	15.0	9.5	*5*
Heater and thermostat	9.9	12.8	*15*	6.4	5.2	*10*
Pressure control	28.5	22.9	*7*	13.3	7.3	*8*
Drain system	12.0	18.4	*11*	11.8	6.3	*15*
Spray arm	–	–	*-*	7.3	7.7	*7*
Pump filters	–	–	*-*	–	8.0	*1*
Drive belt	13.8	21.8	*8*	–	–	*-*
Drum and tub	–	8.1	*11*	–	–	*-*
Other	16.0	13.0	*10*	8.6	12.6	*10*

a Number of diagnosed appliances where the client was able to specify the age of the appliance.

The average age of a repaired washing machine is less than 10 years old in three cases: foreign objects detected, pumps, heaters and thermostats. Looking at the unrepaired appliances, however, the critical components are again drum and tub. The average age of a repaired dishwasher is less than 10 years old in four cases: heaters and thermostats, pumps, electronics and the remaining category “others”. Looking at the unrepaired appliances, however, the critical components are the drain system, and the heaters and thermostats. Looking at the specific datasets of the 2016 database, these represent the recurring failure modes, namely the components more prone to fail, before the appliance reaches the age of 10. The failure modes observed in this subset of data were generally in line with what was observed in the 2009–2015 database.

The fact that repair was often considered too expensive by the clients of the repair centre is the main reason to conclude the average service life of an appliance and to proceed with its replacement. For both washing machines and dishwashers, the median (50^th^ percentile) product age of an unrepaired appliance, due to a consumer choice, is about 10 years. While for dishwashers, it may seem that products older than 10 years are less likeable to be repaired, this is not the case for defective washing machines, even though distribution outliers can be found for appliances older than 20 years. Further analysis is needed to understand how reparability of household appliances can be improved, given that in most of the cases repair is technically possible, but it may become impossible when spare parts for old appliances are no longer available.

## 5 Discussion

### 5.1 Discussion on results

Recurring failure modes were often ascribed to defective components, in particular pumps, electronics, valves and doors for dishwashers; electronics, shock absorbers and bearings, doors, carbon brushes and pumps for washing machines. While most of the defective appliances could have been repaired, the overall cost of spare parts and labour were often judged too high by consumers, who renounced to repair the defective appliance and decided to replace it with a new one.

Results provide a comprehensive overview of repair activities often performed outside the warranty period. We observed that electronic boards play a key role in household appliances, and its presence is growing, thanks to new functions for smart appliances. Unfortunately, when failures occur in this category, the repair rates are quite low. Improved design for disassembly for these parts, and availability of spare parts could facilitate their replacement. On the other hand, the repair of electronics could become more difficult in the future, increasing the complexity of the components and involving also data protection and cybersecurity issues (Polverini et al., 2017). The need of procedures for the deletion of personal data and update of firmware in reused appliance have been also proposed by standard EN 50614, currently under approval (CENELEC, 2018).

We recall that the database of repair services monitored in 2016 (and thus reporting the age of the device) was less populated compared to the 2009–2015 database, and uncertainty may be introduced by a number of factors. However, we could identify which components or recurring failure modes can be often observed when relatively young (e.g. less than 10 years old) appliances are diagnosed. Nonetheless, the limitations identified for this subset of data demand for a further analysis with a more comprehensive database.

Based on the analysis carried out during this work we are able to formulate three main strategies to increase reparability of appliances: 1) to increase the collection rate and storage efficiency of used, but still robust and reliable, spare parts; 2) to improve the design for disassembly of products and components; 3) to improve the availability of information on product disassembly and diagnosis tools for professional repair operators.

Another possible option to increase the reparability of appliances is to extend their legal guarantee, as most failures occur after the 2-year warranty (Boyano Larriba et al., 2017a). A survey of 1,104 end-users of household electrical appliances in England and Wales proved that, on average, consumers are willing to pay over 30% more for longer-life products that are backed by a longer standard guarantee or warranty (WRAP, 2013). This option was for example discussed within the study for the revision of the EU Ecodesign measures for washing machines and washer dryers (Boyano Larriba et al., 2017a), although its concrete application is still under scrutiny. Nonetheless, voluntary extended warranty options already exist and vary across EU countries, manufacturers, models (Boyano Larriba et al., 2017a; Tecchio et al., 2017b).

Moreover, end-users can be better educated to avoid behaviours that may compromise the proper functioning of the appliance (e.g., unlevelled appliance position, incorrect loading, detergent over-dosage, lack of proper maintenance, exclusive use of low-temperature programs, etc.). The relationship between user attitudes and behaviours and failure modes is relevant but this aspect was not investigated. Further dedicated research is recommended on this subject.

### 5.2 Discussion on repair services classification

The classification of repair services, validated by RUSZ, included both information retrieved by professional repair operators and, for the 2016 database, information retrieved through interviews with end-users. This classification system may represent a way to build more comprehensive databases (e.g. including different geographical locations and contexts), but not necessarily more complex (i.e. with limited number of classifiers). However, survey campaigns among other professional repair operators are needed to further validate the classification system. Once validated, this structured common classification system can be used to collect information on repair, to understand how well repair of a given product group reaches several areas (e.g. the EU context, with multiple repair operators per each country).

Results of such a data collection can be then centralized and analysed with statistical metrics, to remove outliers and errors, and to understand trends. Nonetheless, recommendations for data collection (and sharing information) on repair services have to be tailored for specific product groups to provide meaningful information. In order to stimulate the discussion on the parameters to be monitored during the repair services, the development of metrics to support the circular economy and the identification and grouping of the failure modes, we provide our database for the year 2016 as Supplementary material.

## 6 Conclusions

The main objective of this research was to provide quantitative data about frequent failures and insights about the average age of defective dishwashers and defective washing machines.

The research was carried out through a statistical analysis and appropriate visualisation of results of repair services performed by a professional repair operator. The database was initially compiled with about 11,000 repair services on washing machines and dishwasher, adding then 396 cases (255 washing machines and 141 dishwashers) where clients provided additional information concerning the age of the device, the average frequency of use and the previous repair services performed on the appliance. This additional interview was planned in order to study correlations between product failures and product age, but also use intensity and previous repairs.

The exercise performed on the 2016 database allowed us to report a number of final remarks. First of all, beside of the relatively small sample of data, we observed that the frequent failure modes were aligned with what can be observed in the overall database, composed by thousands of records over the period of time 2009–2015. Then, we can highlight the key role played by reparability in determining the service life of a product. From the literature review it was observed that in most of the cases, the reason for the disposal of appliances was a defect, and most of the appliance sales are primarily due to replacements after product failures. Instead, most of the repair services performed by the repair operators were successful, and this results in an extension (that cannot be estimated through the data we retrieved) of the appliance lifetime. Nonetheless, more than three quarters of defective appliances were not repaired because the client judged the overall repair cost too high.

We believe that a more structured data collection on frequent failures and repair rates may be generally useful to understand which components of a given product group are more prone to fail, and which are more challenging to repair. In particular, the components identified to be more unlikely to be repaired represents a discriminating factor when it comes to define when an appliance can be used again or should go to EoL processes. The data collection, if performed at the moment of the initial diagnosis and during the repair service is not necessarily time consuming, and can be done by any repair operators. Statistical analyses may bring the necessary knowledge to identify existing hot-spots, but professional repair operators should record a series of key parameters in a structured way. As a result, a structured knowledge on repair services can be useful (also for EoL operators) to collect spare parts from appliances at the EoL, to be stored for future reuse.

The classification we used to group the datasets on repair operations allowed us to better interpret the results and to draw some considerations on the reparability of the two product groups. Repair operators can collect information about the failure(s) observed and action(s) performed, such as: type(s) of failure, single or multiple failures, reasons not to repair (if unrepaired), type of repair performed (if repaired), use of new or reused spare parts (if replacements occurred). Other complementary information, which may be more subject to uncertainty, can be retrieved by professional repair operators through interviews with end-users. Interviews can include questions on the age of the device at the moment of the failure, the average use frequency and the previous repairs/replacements performed on the same appliance. Although the proposed classification and recommendations were validated by a single professional repair operator, and specifically suited for washing machines and dishwashers, we think these could be extended to other repairers and potentially applicable to other product groups. Additional research on this aspect is needed.

The outcomes of this work highlight the importance of product reparability to extended the lifetime of defective appliances, and lead us to elaborate a structured template for data collection, to be used by repair operators to store and share information about actual data on repair services.

Finally, we remark that this work provides increased knowledge concerning service life of washing machines and dishwashers and is also a good mean to further stimulate the discussions between industries, academia and policymakers on resource efficiency of products groups, as already called upon by Stamminger et al. (2018). Standardisation activities and working groups can be inspired by this analysis to establish common frameworks to collect and process actual data on repair activities, so to monitor the progress and contribution of this practice to the circularity of materials.

## Disclaimer

The information and views set out in this article are those of the authors and do not necessarily reflect the official opinion of the European Union. Neither the European Union institutions and bodies nor any person acting on their behalf may be held responsible for the use which may be made of the information contained therein.
